# Deubiquitinases in muscle physiology and disorders

**DOI:** 10.1042/BST20230562

**Published:** 2024-05-08

**Authors:** Cyriel S. Olie, Darragh P. O'Brien, Hannah B.L. Jones, Zhu Liang, Andreas Damianou, Ilknur Sur-Erdem, Adán Pinto-Fernández, Vered Raz, Benedikt M. Kessler

**Affiliations:** 1 Department of Human Genetics, Leiden University Medical Centre, 2333ZC Leiden, The Netherlands; 2 Target Discovery Institute, Centre for Medicines Discovery, Nuffield Department of Medicine, University of Oxford, Oxford OX3 7FZ, U.K.; 3 Chinese Academy for Medical Sciences Oxford Institute, Nuffield Department of Medicine, University of Oxford, Roosevelt Drive, Oxford OX3 7FZ, U.K.; 4 Nuffield Department of Women's and Reproductive Health, University of Oxford, Women's Centre, John Radcliffe Hospital, Oxford OX3 9DU, U.K.

**Keywords:** deubiquitinase, muscle atrophy, muscle regeneration, myopathies, neuromuscular disorders, ubiquitin

## Abstract

*In vivo*, muscle and neuronal cells are post-mitotic, and their function is predominantly regulated by proteostasis, a multilayer molecular process that maintains a delicate balance of protein homeostasis. The ubiquitin-proteasome system (UPS) is a key regulator of proteostasis. A dysfunctional UPS is a hallmark of muscle ageing and is often impacted in neuromuscular disorders (NMDs). Malfunction of the UPS often results in aberrant protein accumulation which can lead to protein aggregation and/or mis-localization affecting its function. Deubiquitinating enzymes (DUBs) are key players in the UPS, controlling protein turnover and maintaining the free ubiquitin pool. Several mutations in DUB encoding genes are linked to human NMDs, such as *ATXN3*, *OTUD7A*, *UCHL1* and *USP14*, whilst other NMDs are associated with dysregulation of DUB expression. USP5, USP9X and USP14 are implicated in synaptic transmission and remodeling at the neuromuscular junction. Mice lacking USP19 show increased maintenance of lean muscle mass. In this review, we highlight the involvement of DUBs in muscle physiology and NMDs, particularly in processes affecting muscle regeneration, degeneration and inflammation following muscle injury. DUBs have recently garnered much respect as promising drug targets, and their roles in muscle maturation, regeneration and degeneration may provide the framework for novel therapeutics to treat muscular disorders including NMDs, sarcopenia and cachexia.

Ubiquitin is a small protein of 76 amino acids that is highly conserved in eukaryotes [1]. The post-translational modification of protein substrates by ubiquitin is key to most cellular processes impacted by proteostasis. Ubiquitination primarily occurs via covalent isopeptide bond formation to lysine residues via a cascade of enzymatic activities involving the E1 ubiquitin-activating, E2 ubiquitin-conjugating, and E3 ubiquitin–protein ligase enzymes. Notably, ubiquitination can also be facilitated through ester bond formation on serine and threonine, and thioester linkages to cysteines [1]. Next to targeting substrate proteins, carbohydrates, lipopolysaccharides and ribosyl-moieties can also be ubiquitinated, of which the latter is predominantly involved in host-pathogen interactions [2,3]. Through its involvement in the ubiquitin proteasome system (UPS), ubiquitin itself can also be ubiquitinated, forming polyubiquitin chains that tag misfolded, defective or redundant proteins for degradation by the proteasomal system. In contrast with E3 ligases, deubiquitinating enzymes (DUBs) remove ubiquitin moieties and thereby counteract ubiquitination signaling. The widespread nature of ubiquitin-dependent biological processes is reflected by the ∼700 known E3 ligases, and the ∼100 DUB species. DUBs are divided in different families based on distinct mechanisms of action, including ubiquitin-specific proteases (USPs), ubiquitin C-terminal hydrolases (UCHs), ovarian tumor proteases (OTUs), JAB1/MPN/Mov34 metalloenzyme, Machado–Joseph deubiquitinases (MJDs), motif interacting with Ub-containing novel DUB family, and zinc finger containing ubiquitin peptidase 1 (ZUP1) [4,5].

Regulation of proteostasis by the UPS is essential for tissue maintenance, in particular for those of post-mitotic cells, such as neurons and muscle fibers. Over 10% of E3 ligases are implicated in common and rare neurological disorders [6]. In skeletal muscles, two E3 ligases, TRIM63 and FBXO32, also known as MuRF1 and Atrogin-1, regulate muscle atrophy [7], a key pathological feature in muscle degeneration and muscle wasting [8–10]. Here, we surveyed most recent literature in addition to public databases for information on the role of DUBs in a diverse range of biochemical pathways which have been implicated in muscle physiology, in neuromuscular disorders (NMDs) and specifically in muscle wasting. This is particularly timely, as DUBs are emerging as attractive drug targets [11], offering potential inroads into novel therapeutics for NMDs, including myopathies and muscular dystrophies as well as cachexia and age-associated wasting as seen in sarcopenia.

## Ubiquitin processing is linked to muscle biology and physiology

Skeletal muscle, the largest tissue in vertebrates by mass, provides stability and facilitates mobility and balance of the skeleton. All are vital for normal daily functioning. Muscle contraction is voluntarily controlled by motor neurons connecting the central nerve system to nerve terminals and neuromuscular junctions (NMJs) at the muscle plasma membrane. A wide range of movements are accommodated through the activity of over 600 different skeletal muscles which vary in size, shape, architecture, and molecular composition [12,13]. Myofibers are characterized by the sarcomeric protein architecture that facilitates contraction and stability. Myofibers are long, multinucleated cells that are formed during embryo development, but become post-mitotic after birth. Muscle degeneration is characterized by loss of muscle mass (atrophy) and loss of contraction, together negatively affecting mobility and stability in both acute and chronic dystrophy. Repair of damaged muscles is through regeneration, by which muscle stem cells proliferate and fuse into new myofibers. The UPS plays a pivotal role in controlling protein homeostasis in myofibers. Out of the E1, E2 and E3 ligases, it is chiefly the E3 ligases that are implicated in muscle atrophy (reviewed in [14–16]). In contrast with E3 ubiquitin ligases, the role of DUBs in skeletal muscle biology, physiology and muscle disorders is less well-understood [17]. Age-associated loss of muscle mass is accompanied by an up-regulation of many DUBs [18], suggesting multiple roles for DUBs in muscle physiology and pathology. A genetic screen in muscle cells indicates that dysregulation of several DUBs including USP7, USP10, USP13, USP16 and USP18 expression abrogates muscle cell differentiation [19]. The expression profile of four muscle related USPs (USP10, USP14, USP19 and USP45) also differs between fast- and slow-twitch myofibers [20]. For most of these DUBs, their molecular role in the context of muscle biology remains obscure.

## NMDs involving DUB pathogenic variants or aberrant expression associated with muscle integrity and function

DUB mutations associated with muscle pathology are often linked to neurological disorders and inflammation [21,22]. For instance, a biallelic loss of *OTUD7A* causes severe muscular hypotonia associated with intellectual disability and seizures in humans [23] (Table 1). In a separate study, *Otud7a* knockout mice displayed muscle hypotonia, impaired dendritic spine density, and reduced glutamatergic synaptic transmission [39]. Although these studies highlight the importance of OTUD7A with regard to neuromuscular transmission, which likely underlies muscle weakness, the exact molecular mechanisms behind this remain to be determined. Interestingly, Garret et al. [40] found that mutated *OTUD7A* impairs proteasomal activity by regulating proteasomal assembly in a yet unknown manner. Nevertheless, this could indicate that muscle weakness in pathological conditions caused by a *OTUD7A* mutation could, in part, be a result of imbalanced proteostasis.

**Table 1.** Most common DUB pathogenic variants associated with human disease linked muscle pathology

Family	DUB	Human/mouse pathology	Muscle disease trait	References
UCH	*UCHL1* Loss of function variants Deletions 2-223/25-223/insertion 52L > LL E7A I93M R178Q A216D	Processing of ubiquitin precursors and ubiquitinated protein substrates. Its absence impairs the synaptic transmission at the NMJ, inducing profound structural defects at the presynaptic nerve terminals and muscle denervation. Spastic paraplegia 79–autosomal recessive (SP79-AR)	Neurodegeneration Spastic paraplegia 79A Muscle denervation Difficulty with balance, weakness and stiffness in the legs, muscle spasms	[24,25,26]
USP	USP9X FAF-X	Antagonizes ubiquitin-mediated proteolysis, preventing protein degradation and controlling synapse development. Defects in the synaptic transmission at the NMJ	Defects in the synaptic transmission at the neuromuscular junction	[27] [28]
USP	*USP14* 4 bp deletion 233_236delTTCC; p.Leu78Glnfs*11	Crucial for synaptic development and function at the NMJ. Its catalytically inactive form causes developmental deficits in the NMJ structure and synaptic transmission. Its loss causes presynaptic defects	Severe tremors, hind limb paralysis, and postnatal lethality Muscle wasting, cachexia	[29] [30] [31]
USP	USP18 *USP18* truncation at 652C > T (N218)	Pseudo-TORCH syndrome (PTS) microcephaly, enlarged ventricles, cerebral calcification due to severe interferonopathy Mutations linked to multiple sclerosis susceptibility	Muscle spasms, stiffness and weakness Bradycardia Muscle differentiation	[32] [19]
USP	USP19	*Usp19* KO mice have lean muscle phenotype and less myofiber atrophy	Muscular atrophy Muscle wasting, cachexia	[33] [34] [31] [35] [36]
OTU	*OTUD7A/Cezanne* 15q13.3 deletion including the OTUD7A locus, frameshift OTUD7A variant c.1125del, p.(Glu375Aspfs*11)	Intellectual disability, and seizures	Severe muscular hypotonia	[23]
MJD	ATXN3 polyglutamine expansion in ataxin-3 (ATX3; MJD1)	Machado–Joseph disease (MJD), autosomal-dominant neurodegenerative disorder Affects Ca^2+^ signaling	Muscle spasticity	[37,38]

Mutations recorded in DUBs and their associations with pathologies in humans are described. DUB knockout mice and other organisms also show phenotypes including muscle specific traits.

Abbreviations: ATXN, ataxin; DUB, deubiquitinase; OTU, ovarian tumor domain containing protease; MJD, Machado–Joseph-disease; UCH, ubiquitin C-terminal hydrolase; USP, ubiquitin specific protease; NMJ, neuro-muscular junction; FAF, fat facets (fly).

Homozygous missense and splice-site mutations in *UCHL1* have been reported in cases with spastic paraplegia and progressive ataxia (Table 1). Interestingly, genome screening illuminated different frame-shift or in-frame shifts that are predicted to cause a loss-of-function. A p.Glu7Ala and p.Ile983Met missense mutation caused reduced binding affinity and consequently a lowered catalytical activity of <10% and ∼50% as compared with wild-type *UCHL1* [24,25]. These loss-of-function mutations often lead to nerve fiber loss, axonal swelling, degeneration of the spinal cord, and impaired neuromuscular denervation and synaptic transmission. Interestingly, one case showed a heterozygous variant with a missense mutation p.Ala216Asp, leading to an insoluble protein variant, and a p.Arg178Gln mutation that showed a four-fold increased hydrolytic activity [24,25]. The maintenance of the NMJ structure and function is also associated with UCHL1, as *Uchl1* knockout mice develop selective motor neuropathy [41,42]. UCHL1 also regulates lipid and perilipin 2 levels [43] and mitochondrial oxidative activity in skeletal muscles [44]. However, a mode of action of UCHL1 remains unexplored in these KO studies. Guillain–Barré syndrome (GBS), a condition affecting the myelin sheath of nerves, leading to muscle weakness and sometimes paralysis, has been linked with altered UCHL1 levels. Higher levels of UCHL1 were detected in the cerebrospinal fluid of GBS patients, when compared with controls, with a possible association between UCHL1 levels and disease severity [45]. Furthermore, UCHL1 has been found to be significantly higher in the plasma of Parkinson's disease (PD) patients at moderate stages of disease, when compared with early-stage counterparts and healthy controls [46], reflecting a possible correlation with PD pathology [47]. Altogether, it appears that several NMDs are characterized by a change in UCHL1 expression levels, including spinal muscular atrophy (SMA) [48]. However, a positive correlation was found between increased UCHL1 levels and total protein levels in GBS patients [45], indicating that any interpretation of expression levels should be interpreted with caution. Interestingly, UCHL1 has been found to bind the proteasome, and in case of point mutations, such as I93M, C90S and E7A, that have reduced hydrolytic activity, UCHL1 binds the 20S core subunit of the proteasome and negatively affects its activity [49]. The effect of various mutations and deletions in human UCHL1 gene on, for instance, the mitochondrial respiration and proteasomal activity, suggests that both the catalytic and scaffolding functions of UCHL1 are of importance in muscle biology. The study of UCHL1 pathogenic variants have contributed to the understanding of its function in multiple cellular processes, which is key to revealing its mode of action in disease mechanisms.

Spinocerebellar ataxia type 3, also known as Machado–Joseph disease (MJD), is a neurodegenerative disorder caused by a polyglutamine-coding CAG trinucleotide repeat expansion in the MJD domain protease subfamily member ATXN3 (Table 1) [37,50]. Mechanistically, it is not yet fully understood how the ATXN3 variants contribute to ataxia. However, next to nuclear aggregation of mutated ATXN3, it was found that mutated ATXN3 directly binds InsP_3_R1, a calcium channel, causing it to open more easily, leading to aberrant calcium signaling, which contributes to pathogenicity [38,51]. ATXN3 also interacts with VCP (/p97), an ATPase complex, that thereby facilitates the deubiquitination of BECN1 by ATXN3 [52]. Interestingly, reduced proteasomal degradation due to mutations in p97 have been found in the muscle wasting phenotype of amyotrophic lateral sclerosis (ALS) and cachexia, suggesting an important role for ATXN3 and the p97 complex in muscle physiology [53].

SMA is caused by a mutation in the survival motor neuron 1 gene (*SMN1*), which results in low levels of SMN protein, motor neuron degeneration, and ultimately, to lethality in infants. USP9X stabilizes SMN protein by a direct removal of ubiquitin moieties, which might prove to be an interesting target. However, whereas USP9X deubiquitinates wild-type SMN protein, its mutated form is unaffected by USP9X, causing its rapid degradation, and thereby contributes to impaired neuronal growth and development [27]. Elucidating why USP9X does not target mutated SMN protein could prove a valuable avenue in treating SMA, possibly for adult-onset disease. Aberrant morphology of NMJs has been found in *Drosophila* with USP5 mutations and is thought to be linked to altered ubiquitin homeostasis through ubiquilin, a ubiquitin-like protein [54] (Table 1). Nevertheless, a role for USP5 does not seem to exclusively affect NMJs [55].

ALS, also known as motor neurone disease (MND) or Lou Gehrig's disease, is a rare, progressive, and fatal type of degeneration of the spinal cord and motor neurons that control voluntary muscles [56]. Cases of ALS are primarily sporadic, but hereditary forms also exist. Components of the UPS play a pivotal role in ALS [57]. A key hallmark of ALS is the pathogenic deposition of TAR DNA-binding protein 43 (TDP-43) in the spinal cord and brain of both sporadic and familial cases [58]. Ubiquitinated TDP-43 is enriched in ALS brain inclusions [59], which is likely due to the co-ordinated action of UBE2E3 conjugation and either Parkin, VHL/CUL2, Znf179, or Praja 1 ligation [57,60]. Counteracting this, several DUBs have been shown to regulate TDP-43 protein turnover and stress granule clearance, including CYLD [61], USP5/13 [62], USP7 [63], USP8/UBPY [60], USP10 [64], USP14 [65], and USP47 [66]. Moreover, a rare variant of CYLD has been found in a cohort of Chinese ALS patients [67]. Finally, UCHL1 has been proposed as an ALS candidate biomarker in CSF and serum [68,69]. Although these events may affect skeletal muscle indirectly, targeting these DUBs might lead to alleviation of the muscle pathology.

In contrast with NMDs, the role of DUBs in myopathies is less explored. A transcriptomic study of degenerated muscles of oculopharyngeal muscular dystrophy (OPMD) patients and OPMD animal models showed significant dysregulation in DUB expression. OPMD is a late-onset myopathy, caused by an expansion mutation in *PABPN1* that leads to nuclear aggregation of PABPN1. The reduced functional levels of PABPN1 and dysregulated DUB expression implies a role for the UPS [70]. Reduced PABPN1 levels lead to muscle atrophy by direct regulation of *FBXO32* level [71]. PABPN1 has also been shown to directly regulate the transcript levels via alternative polyadenylation site usage of PSMD14/POH1, a metalloprotease that is part of the 19S proteasome that is directly involved in the degradation of polyubiquitinated proteins [70,72]. Another DUB that is associated with the proteasome, USP14, has been implicated in muscle atrophy due to increased expression, but its functional role remains unclear [73]. USP14 has been shown to be essential for the maintenance of synaptic ubiquitin levels and the development of NMJs [29]. A 4-bp biallelic deletion induced loss of USP14 leads to distal arthrogryposis (multiple congenital contractures) in human individuals that is characterized by abnormal development and function of skeletal muscles [30]. Mouse models with no or reduced USP14 expression levels show neurological and muscular abnormalities similar to humans, supporting the notion that USP14 plays a crucial role in development of the neuromuscular system [74,75]. Together, several ubiquitin E3 ligases, DUBs and the biogenesis of the 26S proteasome, have been demonstrated to be involved in enhanced degradation of muscular proteins [31].

USP19 has been shown to be overexpressed in mammalian skeletal muscle in a plethora of degenerative disorders associated with muscle atrophy, including fasting and caloric restriction, diabetes, cancer, and smoking [76]. USP19 knockout mice are viable, and in response to fasting exert less muscle wasting as compared with WT littermates. Lower levels of brown and white adipose tissue have also been found [35,36]. In skeletal muscles of patients with lung and gastrointestinal cancers, a potential link is observed between the expression of USP19, TRIM63 and FBXO32 mRNA, further strengthening the link between USP19 and human muscle wasting [77]. Mechanistically, a functional role linking USP19 activity and expression to diet-induced insulin responsiveness, glucocorticoid signaling, adipogenesis, but also fat and muscle distribution has been demonstrated in mice [36], although the exact molecular details remain to be determined.

Links to insulin responsiveness and skeletal muscle mass have also been demonstrated for USP21 [78]. Removal of USP21 resulted in an increased mitochondrial activity in skeletal muscle, leading to promotion of an oxidative myofiber phenotype, and inhibition of obesity and type 2 diabetes [78]. This phenomenon could be reversed by overexpression of this DUB. Similarly, in skeletal muscles, USP21 is up-regulated in individuals with obesity, and correlates with fasting glucose levels in animals on a high-fat diet [78]. In skeletal muscle, USP9X has been proposed to play a key role in the anti-diabetic effect of calorie restriction through its increase and stabilization of AMPKα2 receptors [79]. Altering the expression levels of USP21 and USP9X have therefore been suggested to have therapeutic potential in the treatment of obesity and impaired insulin sensitivity, which are both associated with muscle physiology.

As introduced earlier, links have also been made between NMDs and the levels of DUBs detected in patient biofluids and tissues. For instance, multiple sclerosis (MS), an autoimmune disorder leading to muscle spasms, stiffness and weakness, has been linked to changes in USP18, TNFAIP3 (A20) and USP16 expression levels. Low levels of *USP18* mRNA were detected in the peripheral blood mononuclear cells of patients with relapsing-remitting MS (RRMS), which could possibly lead to enhanced TNF and type-I interferon (IFN) signaling as these DUBs are major negative regulators of immune signaling [80]. A reduced expression level of TNFAIP3 is observed in monocytes of RRMS patient [81], whereas an increased expression of TNFAIP3 has been found in the lesions of post-mortem brain tissue of MS patients [82]. Also, increased expression of USP16 was recorded in CD3^+^ T cells of MS patients [83]. Furthermore, two USP18 polymorphisms have been associated with MS susceptibility [84] (Table 1). USP18 inactivating mutations in human individuals lead to severe type 1 interferonopathies through a lack of USP18-mediated negative regulation of the IFN signaling and thereby causing severe chronic inflammation, which predominantly affects the CNS, but also other organ systems including the heart (bradycardia) [32].

Taken together, human mutations in DUBs, knockout animal models or their altered expression profiles affecting skeletal muscle pathology and myopathies are often linked to complex phenotypes, predominantly associated with NMDs, in addition to inflammation, cachexia and cancer.

## DUBs in muscle injury and regeneration

### Inflammation

**Figure 1.**
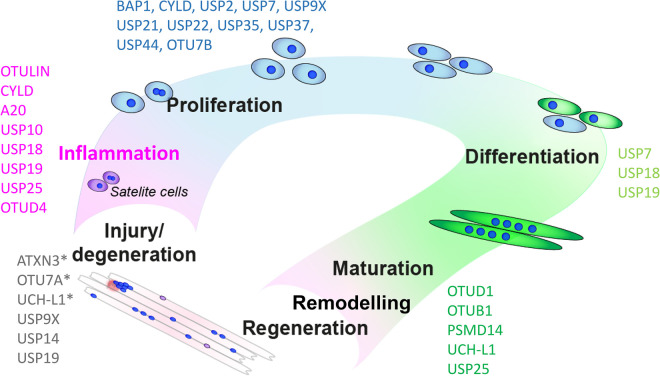
DUBs associated with muscle physiology and pathology. Deubiquitinases (DUBs) have been linked to various stages of muscle development, homeostasis and neuromuscular disorders affected by mutations (indicated with an asterix *, see also Table 1) and altered expression profiles. DUBs associate with diverse stages of muscle physiology, such as muscle injury/damage, muscle inflammation (e.g. myositis), proliferation, myogenic differentiation, muscle regeneration, remodeling and maturation. DUBs highlighted in black (bold) have been studied in skeletal muscles directly. It is likely that DUBs highlighted in gray might have an indirect effect on skeletal muscle physiology and pathology. See text for details.

Skeletal muscles have a strong capacity to regenerate injured tissue via a cascade of interconnected events [85,86] (Figure 1). In damaged and diseased muscle tissues, an inflammatory response is activated to mediate regeneration and tissue repair. This inflammatory response in muscles involves the secretion of cytokines, such as IFNs, IL-1β and TNF-α, resulting in the attraction of immune cells, macrophages, neutrophils, T-cells and B-cells [85]. This immune response leads to the removal of damaged cells, after which new migrating cell populations will repair the damaged tissue regions [86]. However, in case of an excessive or a chronic inflammatory response as seen in inflammatory myopathies, regeneration fails and results in muscle wasting instead. In other muscular dystrophies, such as Duchenne muscular dystrophy (DMD), a prolonged inflammatory response has also been shown to exacerbate muscle damage, impair regeneration and associate with disease severity [87,88]. Inflammatory myopathies (myositis), such as polymyositis, inclusion body myositis and dermatomyositis, often involve excessive cytokine [89] and type 1 or 2 IFN expression that lead to muscle weakness (reviewed in [90]). Cytokine levels are relevant in the diagnostics and differentiate between various types of myositis [91,92]. IFN production and the respective response pathways are tightly regulated by ubiquitination and deubiquitination events, and as a consequence, regulatory functions for several DUBs have been described. For example, TNFAIP3, CYLD, USP3, USP4, USP15, USP25, and OTUD5 deubiquitinate key components of the IFN production pathway, including RIG-I, TRAF2, TRAF3, TRAF6, RIP1, and TRIF (reviewed in [93,94]). As for IFN response pathways, USP18 attenuates the type 1 IFN response via a negative feedback loop, and it is the main enzyme involved in the catalytical removal of the interferon stimulated gene 15 (ISG15) a di-ubiquitin analogue, from substrate proteins [95].

IRAK signaling is part of the TLR/MYD88 cascade which is controlled by the DUBs OTULIN, OTUD4, CYLD, TNFAIP3, USP19 and USP25, all of which edit linear and K63-linked ubiquitin chains [96,97]. The MYD88 cascade has been found to mediate muscle wasting in cachexia as well in a model for muscle denervation, highlighting its importance in muscle physiology [98,99]. Furthermore, a long non-coding RNA, referred to as INKILN (inflammatory MKL1 interacting lncRNA), can induce vascular smooth muscle inflammation via scaffolding of MKL1 (myocardin-like protein) and USP10 [100]. Similarly, the long-noncoding RNA Atrolnc-1 that binds to A20, can induce muscle wasting in CKD mice [101].

IL-1β, another cytokine essential for muscle regeneration [102], is initially expressed by activated monocytes and macrophages in response to priming signals from receptors, such as Toll-like receptors (TLRs) or nucleotide-binding oligomerization domain-like receptors (NLRs). It is then processed and activated through the inflammasome system, a critical process in muscle inflammation that is tightly regulated by several DUBs including USP7/47, BRCC3, TNFAIP3, STAMBP, UCHL1 [103] and other members of the UCH family (reviewed in [104]). Interestingly, NLRP3 removal attenuates inflammation induced skeletal muscle atrophy [105]. Down-regulation of NLRP3 and IL-1β levels by adiponectin treatment protect dystrophic mice muscles from excessive inflammation, which ultimately might prove therapeutically relevant for DMD patients [106]. IL-1β signaling was also found to be promoted by IRAK1 induced phosphorylation of USP20 in vascular smooth muscle cells and vascular inflammation [107,108].

USP19 attenuates the TNF-α– and IL-1β–triggered inflammatory response via deubiquitination of TGF-β-activated kinase 1. Mice lacking USP19 were shown to have increased levels of cytokines and elevated inflammatory responses [109]. Taken together, DUBs are essential in regulating cytokine expression and signaling, and thereby ultimately in regulating the balance between a necessary immune response for muscle tissue regeneration or an excessive inflammatory response that causes more damage to the muscle. Despite this essential role and thus also therapeutic potential, the role of DUBs on inflammation of skeletal muscles remains poorly studied (Figure 1).

### Proliferation and myogenic fusion

Cytokines that regulate the progression of removal of damaged tissue can activate quiescent satellite cells (SCs). The activated SCs will provide new cells for tissue regeneration (Figure 1). Muscle regulatory transcription factors (MRFs) regulate the myogenic program. MYF5 and MYOD stimulate muscle cell proliferation and subsequent expression of MYF6 and myogenin initiate the terminal differentiation into multinucleated myofibers. The dynamic expression of these MRFs is crucial and a function for DUBs is becoming more evident. A DUB siRNA screen using myogenic fusion in tissue culture as a readout has revealed several DUBs that affect cell proliferation, differentiation and maturation [19]. In this study, USP7, USP10, USP13, USP18, USP45, UCHL1 and SENP2 were all found to reduce myogenesis and may therefore all be essential for the switch from proliferation to differentiation [19]. More generally, many DUBs, such as BAP1, CYLD, USP2, USP7, USP9X, USP21, USP22, USP35, USP37, USP44 and Cezanne/OTUD7B, have been implicated in cellular proliferation across different stages of the cell cycle [110]. As these DUBs are ubiquitously expressed, they may also affect myocyte and cardiomyocyte proliferation, by controlling the turnover of MyoD [111] and TATA-binding protein, another transcription regulator that is stabilized via direct deubiquitination by USP10 [112]. A second regulator of muscle specific transcripts involved in muscle cell differentiation, Myocyte-specific enhancer factor 2A, was found to be targeted by SENP2. SENP2 promotes myostatin expression and was found to inhibit muscle cell differentiation [113]. Down-regulation of USP7 or pharmacological inhibition impairs muscle differentiation by affecting myogenin stability, thereby enhancing SC myogenic progression [114].

USP19 is perhaps one of the most studied DUBs in pathological conditions characterized by muscle atrophy, but also with regards to muscle cell differentiation and maturation [34,115]. USP19 may interfere with hormone homeostasis in skeletal muscle [116], possibly through a 17β-estradiol (E2) and (o)estrogen receptor (ER)-dependent feedback loop [117]. Only the ER-localized isoform of USP19 (USP19-ER) modulated myoblast fusion as well as the expression of myogenin and myofibrillar proteins in a USP19 catalytic activity-dependent manner [33,118]. This may involve ER related functions of USP19 including stabilization of endoplasmic-reticulum-associated protein degradation substrates [119–121] and the HRD1 ubiquitin E3 ligase [120]. Also, USP19 activates NRF1 (encoded by *Nfe2l1*), thereby altering glucose deprivation sensing, cholesterol abundance, proteasomal inhibition and oxidative stress [122]. Underscoring its importance in muscle physiology, the Almac Group have recently described the first-in-class inhibitor of USP19 for the treatment of cancer-induced muscle atrophy [121].

USP18 was recently identified to be a key regulator of muscle cell proliferation, differentiation and maturation [19]. USP18 enzymatic function typically regulates the fate of proteins modified by ISG15 in the context of innate immune activation by removing ISG15 from protein substrates. However, in muscle cells, USP18 may have an additional role, independent from ISG15, in regulating the timing of differentiation and subsequently maturation. The way USP18 achieves this is perhaps through altering the expression of myogenic transcription (co) factors. In this model, a predominantly nuclear isoform of USP18 seems to regulate gene transcription in a non-canonical fashion. Similar functions of USP18 have been previously described in the context of cancer and involve the association with STAT2, IRF9, and the formation of an active transcription complex that binds to specific IFN-responsive elements [123,124]. Inhibition of either USP18 catalytic activity or scaffolding functions [125] may result in different outcomes as the catalytic activity predominantly affects the removal of ISG15 (deISG15ylation) of protein substrates, most of which are ISGs, whereas scaffolding negatively regulates IFN signaling, thereby possibly affecting cell viability [126–128]. Although future research is required to fully appreciate how USP18 regulates muscle differentiation and maturation, USP18, and its nuclear isoform in particular, might represent an attractive therapeutic target [83].

### Remodeling and functional recovery

Ubiquitin-proteasome mediated proteolysis has been associated with muscle regeneration and remodeling [129]. In particular, the expression levels of DUBs appear to be induced after passive leg cycling in persons with spinal cord injury [130], but also more generally after acute tendon/muscle injury [131] as well as other proteases [132]. Muscle atrophy is observed in muscle wasting, cachexia and muscular diseases such as OPMD. Mechanistically, UPS-associated DUBs may play a role, such as POH1/PSMD14, a DUB metalloprotease associated with the 26S proteasome complex and involved in regulating muscle size/proteostasis via the PI3K-PKB/Akt-mTORC1-FoxO pathway [133]. Generally, a key feature is the development and restoration of synaptic connections to the muscle, which is also regulated by ubiquitin-dependent mechanisms [28]. On the other end of the spectrum, possibly different from skeletal muscles, USP25, UCHL1 and OTUB1 were suggested to attenuate pathological hypertrophy in cardiomyocytes by stabilizing SERCA2a, EGFR and DEPTOR, respectively [134–136]. In addition, OTUD1 and USP15 appear to modulate cardiac myocyte remodeling in the context of heart failure, the former by targeting STAT3 [137,138]. Taken together, DUBs have specific roles in regenerating skeletal muscle mass, in particular in NMJ integrity, maintenance and development.

## Translational opportunities

The ubiquitin system has long been considered an attractive target area for the treatment of muscle atrophies and wasting diseases, with varying degrees of success. Targeting of disease-causing proteins by using small molecules called proteolysis-targeting chimera (PROTAC) protein degraders that molecularly link E3 ligases with those target proteins, has for example been promising with various PROTACs currently in clinical trials [139]. Recent insights into molecular mechanisms now also reveal many DUBs as potential therapeutic targets, with selective small molecule inhibitors in development [11,140]. In fact, USP1 and USP30 inhibitors are currently being tested in the clinics for the treatment of solid tumors and chronic kidney disease, respectively [141–143]. UCHL1 could also prove a promising therapeutic target, since impaired UCHL1 expression and function have been found in various NMDs (Table 1). However, further investigation is required to pinpoint whether UCHL1 catalytical activity or its scaffolding role or both should be targeted. We recently found transient inhibition of USP18 expression to enhance muscle differentiation, and thereby possibly providing a therapeutically interesting target with regards to muscle recovery, after injury. However, the interplay between the type 1 IFN immune response and the newly-described type 1 IFN-independent role of USP18 [19] in muscle cell differentiation should be clarified first, especially in an *in vivo* context. Notably, USP19 inhibition could potentially ameliorate maintenance of muscle mass in muscle atrophy, obesity-linked loss of lean muscle mass [144] and sarcopenia, all of which reflect major comorbidity traits in ageing. Alternatively, as an example, stabilization of critical elements of myogenic transcription, such as MyoD, by DUB activation or recruitment-enhanced deubiquitination may also represent a viable strategy to maintain muscle mass. RESTORACs or deubiquitinase-targeting chimera molecules, in contrast with PROTACs, prevent aberrant protein degradation by selectively recruiting DUBs that remove ubiquitin from the respective target proteins and might thereby be considered for muscle wasting conditions [145,146].

In summary, the pivotal role of the ubiquitin system in various aspects of muscle pathologies, and more recent insights into the role of DUBs in these processes offer potential pharmacological inroads for the design and development of novel therapeutics.

## Perspectives

- *Highlight importance of the field*: human mutations in DUBs, knockouts in animal models or their altered expression profiles all contribute to skeletal muscle physiology and myopathies that are often associated with aberrant neuronal development, NMDs and inflammation.
- *Summary of the current thinking*: the ubiquitin system has long been considered an attractive target area for the treatment of muscle atrophies and wasting diseases. In addition to opportunities for protein degraders, recent insights into molecular mechanisms increasingly reveal many DUBs as potential therapeutic targets, with selective small molecule inhibitors in development.
- *Future directions*: regarding skeletal muscle and muscle-associated diseases, several DUBs including USP14, USP18, USP19 and UCHL1 represent attractive therapeutic modalities, although not only through inhibition, but also activation (restoring function) mechanisms. Preserving muscle mass and function is relevant in the context of myopathies and NMDs, inflammation, cachexia, cancer and ageing (sarcopenia).

## Open Access

Open access for this article was enabled by the participation of University of Oxford in an all-inclusive *Read & Publish* agreement with Portland Press and the Biochemical Society under a transformative agreement with JISC.

## Abbreviations

ALS: amyotrophic lateral sclerosis
DMD: Duchenne muscular dystrophy
DUB: deubiquitinase
ER: (o)estrogen receptor
GBS: Guillain-Barré syndrome
IFN: Interferon
INKILN: inflammatory MKL1 interacting lncRNA
ISG: interferon-stimulating gene
MJD: Machado–Joseph deubiquitinases
MRF: muscle regulatory transcription factors
MS: multiple sclerosis
NMD: neuromuscular dystrophy
OPMD: oculopharyngeal muscular dystrophy
OTU: ovarian tumor
PD: Parkinson's disease
PROTAC: proteolysis-targeting chimera
RRMS: relapsing-remitting multiple sclerosis
SC: satellite cell
SMA: spinal muscular atrophy
TDP-43: TAR DNA-binding protein 43
TLR: Toll-like receptor
UCH: ubiquitin C-terminal hydrolases
UPS: ubiquitin proteasome system
USP: ubiquitin-specific protease
ZUP1: zinc finger containing ubiquitin peptidase 1
